# Gossypiboma

**DOI:** 10.11604/pamj.2015.20.332.6609

**Published:** 2015-04-07

**Authors:** Fouad Hajji, Ahmed Ameur

**Affiliations:** 1 Department of Urology, Mohammed V Military University Hospital, Rabat, Morocco

**Keywords:** Gossypiboma, retained surgical sponge, cotton-based foreign body

## Image in medicine

A 47-year-old woman with a history of nephrolithiasis complicated by chronic renal failure presented with recurrent left flank pain. Nine months ago, she had been subject to an open surgery for left kidney pyonephrosis. She described a short febrile post-operative period; however, there had been no fever for the last eight months. On physical examination, she was afebrile and had a palpable tender mass in the left flank to suggest malignancy. Unenhanced abdominal CT showed a well-encapsulated ovoid heterogeneous mass, located within the left lumbar region (A). The mass resection was done (B). The patient tolerated the procedure and the postoperative period was uncomplicated. Since pathological examination showed retained surgical sponges surrounded by fibrous capsule as foreign body reaction (C), gossypiboma was diagnosed. A gossypiboma refers to a cotton-based foreign body left inadvertently in the human body following a surgical procedure. Gossypiboma is an uncommon condition, diagnosed preoperatively in only one third of all cases. New-onset or a recurrent tumor is the most common differential diagnosis. Gossypiboma should be considered in any postoperative patient presenting with pain, infection, or a palpable mass, particularly in countries where strict surgical protocols may not be in place.

**Figure 1:**
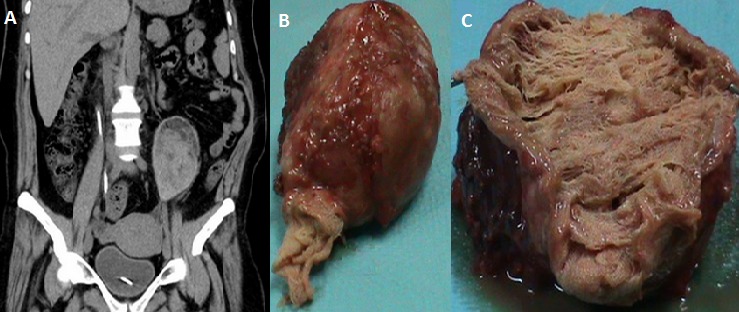
Gossypiboma or retained surgical sponges: A) unenhanced abdominal computed tomography showing a well-encapsulated ovoid heterogeneous mass, located within the left lumbar region; B) resected mass; C) pathological examination showing retained surgical sponges surrounded by fibrous capsule

